# Novel chimeric transcript RRM2-c2orf48 promotes metastasis in nasopharyngeal carcinoma

**DOI:** 10.1038/cddis.2017.402

**Published:** 2017-09-14

**Authors:** Ping Han, Ren-Hui Chen, Fang Wang, Jia-Yi Zeng, Shi-Tong Yu, Li-Hua Xu, Qian Cai, Fa-Ya Liang, Tian-Liang Xia, Zhi-Rui Lin, Qian Zhong, Xiao-Ming Huang

**Affiliations:** 1 Department of Otolaryngology-Head and Neck Surgery, Sun Yat-sen Memorial Hospital, Sun Yat-sen University, 107 Yanjiang West Road, Guangzhou 510120, China; 2 Guangdong Provincial Key Laboratory of Malignant Tumor Epigenetics and Gene Regulation, Sun Yat-sen Memorial Hospital, Sun Yat-sen University, Guangzhou 510120, China; 3 Department of Molecular Diagnosis, Sun Yat-sen University Cancer Center, 651 Dongfeng East Road, Guangzhou 510060, China; 4 Guangzhou Zhixin High School, 152 Zhixin South Road, Guangzhou 510080, China; 5 Department of Hematology, the First Affiliated Hospital of Guangzhou Medical University, 151 Yanjiang Road, Guangzhou 510230, China; 6 State Engineering Laboratory of Medical Key Technologies Application of Synthetic Biology, Shenzhen Second People's Hospital, The First Affiliated Hospital of Shenzhen University, Shenzhen 518035, China; 7 State Key Laboratory of Oncology in South China, Collaborative Innovation Center for Cancer Medicine, Sun Yat-sen University Cancer Center, 651 Dongfeng East Road, Guangzhou 510060, China

## Abstract

Recently, chimeric transcripts have been found to be associated with the pathogenesis and poor prognosis of malignant tumors. Through our preliminary experiment, a novel chimeric transcript called chimeric transcript RRM2-c2orf48 was detected in C666-1, a classical cell line of human nasopharyngeal carcinoma (NPC). Therefore, the objective of this study was to demonstrate the existence and expression of novel chimeric transcript RRM2-c2orf48 and to explore the main functions and mechanisms of RRM2-c2orf48 in NPC. In this study, the expression of RRM2-c2orf48 was evaluated in NPC cells and specimens. Effects of RRM2-c2orf48 on migration and invasive capacities were detected *in* *vivo and vitro*. Moreover, ways in which RRM2-c2orf48 increases the invasive capacities of NPC were explored. As a result, the presence of novel chimeric transcript RRM2-c2orf48 was confirmed in C666-1 by RT-PCR and sequencing, and it was a read-through between RRM2 and c2orf48 through the transcription of interchromosome. Higher expressions of novel RRM2-c2orf48 were detected in NPC cell lines and NPC tissue specimens relative to the controls and its expression was be statistically relevant to TNM staging. High level of RRM2-c2orf48 could increase the migration and invasive capacities of NPC cells, potentially as a result of NPC cell epithelial–mesenchymal transition. RRM2-c2orf48 could also enhance resistance of chemotherapy. In *vivo*, RRM2-c2orf48 could enhance lung and lymph node metastasis in nude mice. These results demonstrate that high levels of RRM2-c2orf48 expression may be a useful predictor of NPC patients of metastatic potency, presenting potential implications for NPC diagnosis and therapy.

Nasopharyngeal carcinoma (NPC) is a form of malignant carcinoma that is endemic in certain regions of the world, especially in Southeast Asia, where the incidence rate of NPC is 20–40 per 100 000 people every year. Although several predisposing epidemiological, genetic and environmental factors have been identified, the exact etiology of the disease remains unknown. Meanwhile, ~85% of patients have regional lymph node metastasis at initial diagnosis,^1^ and an additional 15.7% will develop distant metastasis over the disease’s progression.^2^ Once metastasis is diagnosed, the survival time is typically <15 months.^3^ Therefore, the discovery of novel biomarkers of NPC metastasis would play a critical role in prognostic predictions and could facilitate the development of more personalized therapies for NPC patients.

Over the last few years, studies of chimeric RNAs as biomarkers have been conducted. Many methods, such as next-generation sequencing methods, have been developed to identify biomarkers that are specifically expressed in diseases such as cancer.^4^ A chimeric RNA is an RNA molecule composed of two or more pieces of RNA from different loci that should not be found in the same molecule. Chimeric RNAs are initially identified from transcripts of fusion genes as a result of chromosome translation, inversion or deletion. Studies have indicated that some other RNA chimeras are generated through transcription read-through and splicing. To date, most cancer-related chimeric RNAs have been found primarily in malignancies^5, 6, 7, 8^ in which they have been used to identify particular cancer subtypes and as potentially valuable diagnostic and prognostic biomarkers.^4, 9, 10, 11, 12, 13^ However, chimeric transcripts remain poorly characterized in head and neck malignancies.

In this study, we performed next-generation whole transcriptome sequencing (RNA-Seq) to identify additional molecular diagnostic markers in NPC, and we investigated a set of several highly recurrent chimeric RNAs including chimeric RNA RRM2-c2orf48. We characterized the presence of RRM2-c2orf48 in NPC and noncancerous cell lines. RRM2-c2orf48 overexpression could enhance the migration and invasive capacities of NPC cells. Thus, our results suggest that aberrant chimeric RNA RRM2-c2orf48 is abundant and may be a novel molecular marker of NPC metastasis.

## Results

### Identification of chimeric RNA RRM2-c2orf48 in NPC cell lines

**Figure 1:**
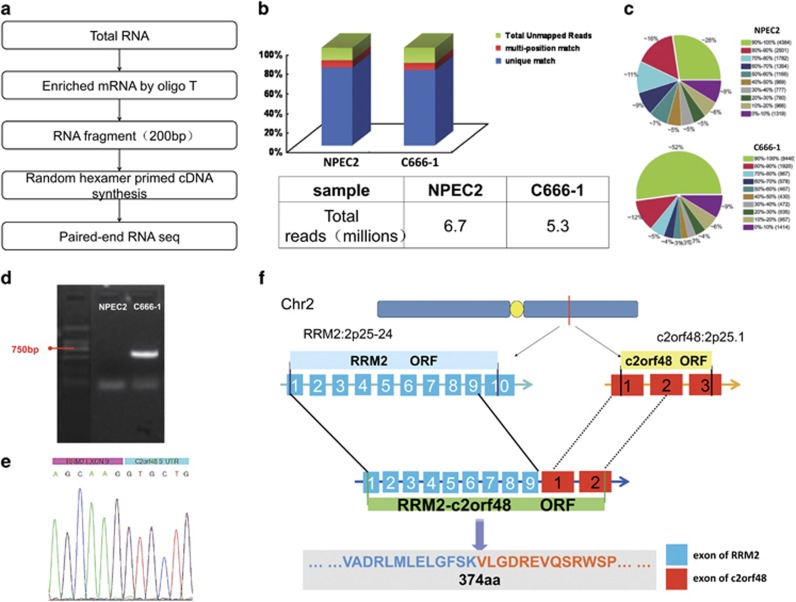
Paired-end RNA-seq data and fusion gene analysis. (**a**) RNA-seq experiment process. (**b**) Data statistics and reads comparison between NPEC2 and C666-1; the unique reads ratio of both templates was >80%. (**c**) The gene coverage results show that the ratio of gene coverage reaching >70% was 55% and 69% in NPEC2 and C666-1, respectively. (**d**) The representative 1% agarose gel image of the amplification of chimeric transcript RRM2-c2orf48 by templates of cDNA from nasopharyngeal carcinoma cell line C666-1 and nasopharyngeal epithelium NPEC2 cells. The specific PCR product of RRM2-c2orf48 is approximately 600 bp in C666-1 but not in NPEC2. (**e**) Sequencing results show the chimeric site between RRM2 and c2orf48 at the mRNA level. (**f**) Formation model of chimeric transcript RRM2-c2orf48: RRM2 is located on chromosome 2p25-24, and c2orf48 is located on 2p25.1. During transcription, read-through occurred between RRM2 exon9 and c2orf48 exon 1. As a result, a new protein with 374 amino acids that was shorter than the wild-type RRM2 was produced (389 amino acids)

Using the paired-end RNA-seq technique, a transcriptone analysis was performed in NPC cell line C666-1 and NPEC2. Using the Illumina GA IIx sequencing platform, 5.3 × 10^7^ reads were attained in C666-1 cells, of which 4.5 × 10^7^ reads (84.34%, 3.1 Gb) could be matched to human reference genomes (hg18), whereas 6.7 × 10^7^ reads were attained in NPEC2 and 5.8 × 10^7^ could be matched to human reference genomes (Figures 1a and c). After a powerful computer cluster analysis of mass data on the sequencing and detection of novel fusion genes was conducted, eight different chimera candidates were found in C666-1 but none in NPEC2. Paired primers were designed across the chimeric sites of these eight chimera candidates(Supplementary Figure 1A). During the procedure of RNA-Seq, poly dT primers were used to enrich mRNA with poly-A tail. Therefore, only transcripts with poly-A tail could be analyzed. Then, PCR amplification was carried out, the product was analyzed via Sanger sequencing, and five of the eight chimeras were identified as positive, including RRM2-c2orf48, which was interchromosome chimeric transcript (Supplementary Figure 1B). The upstream (RRM2) and downstream (c2orf48) areas of RRM2-c2orf48 were positioned in the same chromosome, and the distance between the two genes was found to be <3 MB, showing that the chimeric transcription may form via read-through processes.

### RRM2-c2orf48 validation in NPC cell line C666-1

Primers of chimeric RNA RRM2-c2orf48 were designed in the locations of RRM2 exons upstream and c2orf48 exons downstream. The RT-PCR results show that a single strip could be amplified in a C666-1 template at a length of ~600 bp (Figure 1d). The strip was confirmed as RRM2-c2orf48 by Sanger sequencing. The full-length of chimeric RNA RRM2-c2orf48 was 1125 bp. Our comparative analysis shows that the novel chimeric transcript RRM2-c2orf48 includes the first nine exons of RRM2 RNA, the five non-coding sequences (untranslated regions, UTR), the 1st exon and the partial 2nd exon of c2orf48 RNA (Figure 1e). We speculated that novel chimeric RNA could be translated to a protein with 374 amino acids (Figure 1f).

### Expression of RRM2-c2orf48 at mRNA levels in cell lines and NPC specimens

**Figure 2:**
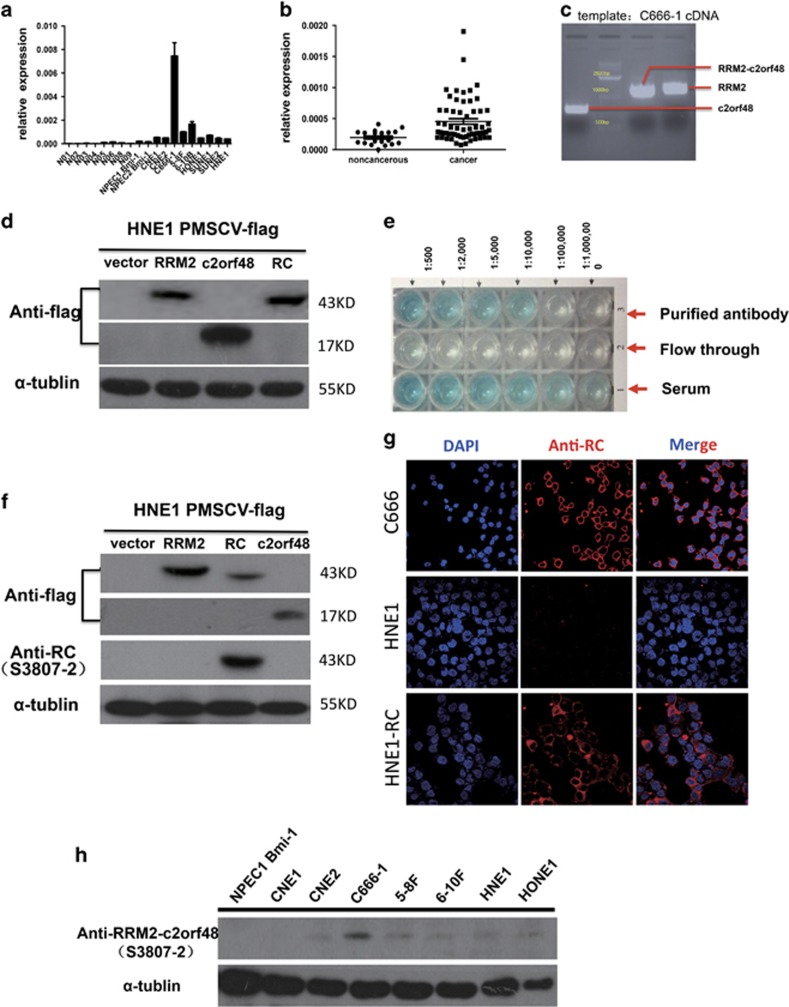
RRM2-c2orf48 expression in NPC. (**a**) The expression level of RRM2-c2orf48 mRNA in nasopharyngeal carcinoma cell lines (CNE1 to HNE1) was higher than that in normal nasopharyngeal epithelium cells (N01–09) and in immortalized nasopharyngeal cell lines (NPEC1-2 Bmi-1) by real-time PCR. (**b**) The expression level of RRM2-c2orf48 mRNA in nasopharyngeal carcinoma specimens was higher than that in the control specimens by real-time PCR (*P*=0.03). (**c**) Amplification of RRM2-c2orf48 and wild-type RRM2, c2orf48 in C666-1 cDNA. Electrophoresis results of 1% agarose gel show a specific band with lengths of RRM2 (1170 bp, nm_001034), RRM2-c2orf48 (1125 bp) and c2orf48 (480 bp, nm_182626), respectively. (**d**) RRM2-c2orf48 (43 kd), RRM2 (45 kd) and c2orf48 (17 kd) expression was detected in HNE1 PMSCV or HNE1 PMSCV-flag-c2orf48/RRM2/c2orf48 cell lines using anti-flag antibodies by western blotting. (**e**) ELISA result of a new synthesized polyclonal antibody against the chimeric region of RRM2-c2orf48. (**f**) The band of RRM2-c2orf48 can be identified from anti-flag and synthesized antibody. (**g**) To confirm the specificity of synthesized antibody, immunofluorescence was used in NPC cell lines HNE1 and C666-1, expressing lower and higher RRM2-c2orf48, respectively. And RRM2-c2orf48 was located in cytoplasm. (**h**) RRM2-c2orf48 protein expression in most of the NPC cell lines was higher than that in NPEC1 Bmi-1

To examine RRM2-c2orf48 expression in mRNA levels of NPC, we performed RRM2-c2orf48 quantitative RT-PCR (qRT-PCR) in NN01–09, immortalized NPECs and NPC cell lines, which include CNE1, CNE2, 6-10B, 5-8 F, HONE1, SUNE1 and HNE1 cell lines. RRM2-c2orf48 has a higher mRNA expression level in NPC cell lines (especially in C666-1) than in immortalized NPECs (Figure 2a). To further examine whether high RRM2-c2orf48 expression can be found in NPC patients, we performed RRM2-c2orf48 qRT-PCR in 60 NPC tissues and 20 noncancerous nasopharyngeal epithelial specimens. As is shown in Figure 1b, the mean expression level in NPC specimens was significantly higher than that in noncancerous nasopharyngeal epithelial specimens (*P*=0.0017, Figure 2b). Meanwhile, wild-type RRM2^14^ and c2orf48 were also higher in NPC specimens and cell lines (Supplementary Figure 2A-B). ACTD result revealed that the mRNA stabilities of RRM2 and c2orf48 was lower in CNE2-RC cell lines compared with those in CNE2-vector cell lines (Supplementary Figure 4).

### RRM2-c2orf48 encodes and leads to the translation of a novel fusion protein

As predicted by Premier Primer 5 software, a 43 kd protein can be translated from chimeric transcript RRM2-c2orf48. To investigate this possibility, we cloned full-length ORF of RRM2-c2orf48 (Figure 2c), tagged it with a flag sequence and transfected it to HEK 293FT cells. A western blotting analysis confirms that RRM2-c2orf48 was indeed translated into a chimeric protein. The molecular weight of the chimeric protein was slightly lower than that of the wild-type RRM2 (45 kd) (Figure 2d). Thus, the chimeric RRM2-c2orf48 may function through its translated protein, which may be an attractive candidate as a useful biomarker for NPC.

### RRM2-c2orf48 expression in cell line and NPC specimen protein levels

Chimeric transcript RRM2-c2orf48 has not been reported on in the literature. The polyclonal antibody against the chimeric region was produced by the China Branch of ProteinTech Group (Wuhan, Hu Bei Province, PR China). The antibody was referred to C-terminal amino acids of RRM2-c2orf48 (VLGDREVQSRWSPGPRGDSTPVREMETNHPPSVRG) and was validated through an ELISA assay (Figure 2e). The band of RRM2-c2orf48 can be identified from a flag antibody or from the new synthesized antibody against RRM2-c2orf48, and demonstrated that the developed RRM2-c2orf48 antibody was effectively RRM2-c2orf48 specific (Figure 2f). As shown in revised manuscript Figure 2g, the developed RRM2-c2orf48 antibody could detect the endogenous RRM2-c2orf48 in C666-1 and exogenous RRM2-c2orf48 in HNE1 RRM2-c2orf48 cells. RRM2-c2orf48 expression was detected in NPC cell lines and NPEC1 Bmi-1. The results show that the expression of chimeric protein RRM2-c2orf48 levels in most NPC cell lines is higher than that in NPEC1 Bmi-1 (Figure 2h).

**Figure 3:**
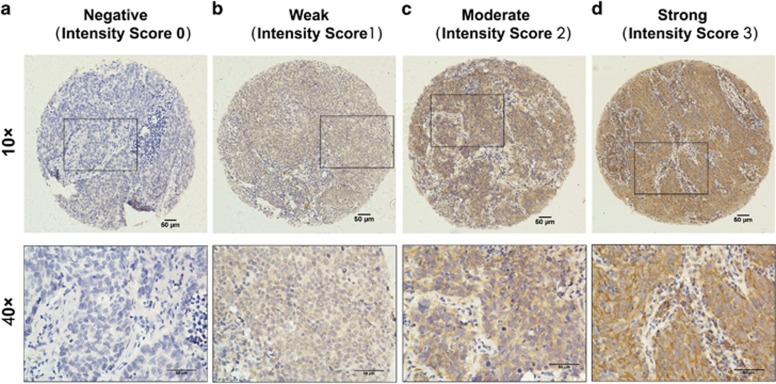
Representative IHC staining of RRM2-c2orf48 in NPC specimens. (**a**) Nearly negative expression of RRM2-c2orf48; intensity level scored as 0. (**b**) Weak expression of RRM2-c2orf48; intensity level scored as 1. (**c**) Moderate expression of RRM2-c2orf48; intensity level scored as 2. (**d**) Strong expression of RRM2-c2orf48; intensity level scored as 3

**Figure 4:**
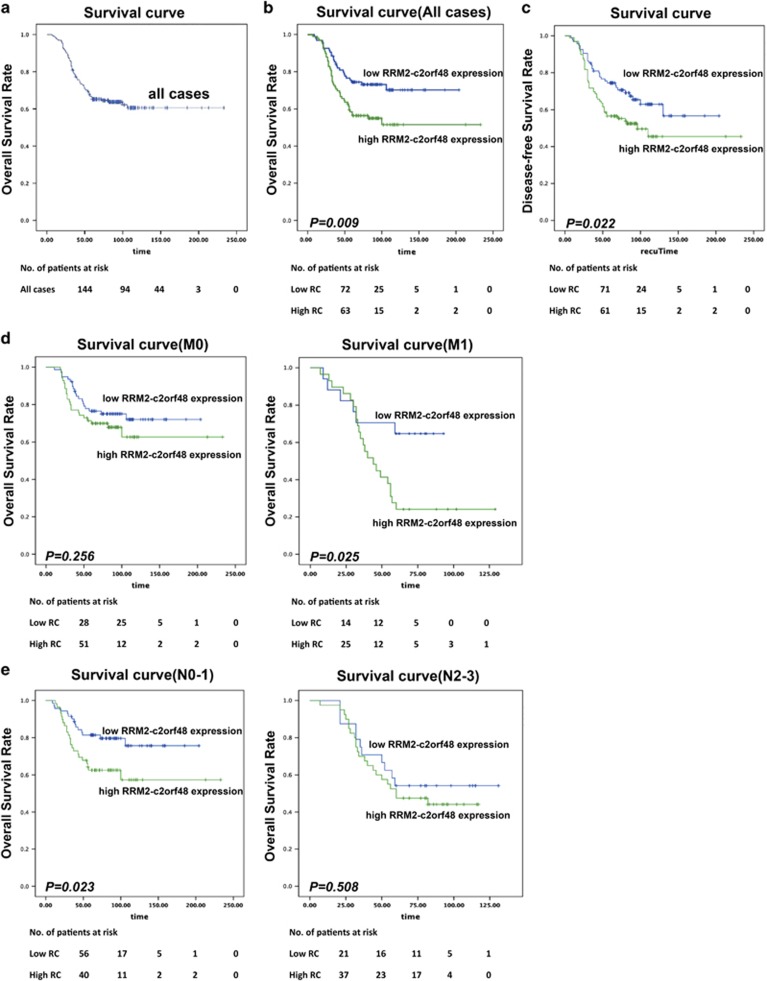
Survival analysis of RRM2-c2orf48 expression in NPC. (Number of patients at different time point was shown below the curve). (**a**) The five-year OS rate of the cohort of 194 NPC patients was measured as 63.4%. (**b**) The Kaplan–Meier and log-rank test analyses show that the cumulative 5-year survival rate reached 54.5% in the high RRM2-c2orf48 expression group, whereas it reached 72.6% in the low RRM2-c2orf48 expression group (*P*<0.01). (**c**) The association between high RRM2-c2orf48 expression and shorter OS was significantly stronger in patients occupying M1 stages than in those occupying M0 stages (*P*<0.05). (**d**) The association between high RRM2-c2orf48 expression and shorter OS was significantly stronger in patients occupying N0–1 stages than in those occupying N2–3 stages (*P>*0.05). (**e**) Disease-free survival (DFS) in patients presenting high RRM2-c2orf48 protein expression (130.3 months) were lower than those in patients presenting low RRM2-c2orf48 expression (141.2 months). *P*=0.022

To examine protein expression levels of RRM2-c2orf48 in NPC tissues, we performed an IHC analysis using a monoclonal antibody against RRM2-c2orf48 in 194 NPC specimens (Figure 3). RRM2-c2orf48 was found to be mainly localized in NPC cell cytoplasms. We analyzed the relationship between RRM2-c2orf48 expression and clinical features of NPC patients. High RRM2-c2orf48 expression levels were observed in 99/194 (51.0%) of the NPC samples. RRM2-c2orf48 expression was significantly associated with the T (*P*=0.001), N (*P*=0.025), and clinical staging (*P*<0.001) in NPC patients. However, no significant correlation was found between RRM2-c2orf48 expression and other clinicopathological features such as patient age, gender, M staging and WHO classification (*P*>0.05) (Table 1). The 5-year overall survival (OS) rate of the cohort of 194 NPC patients was 63.4% (Figure 4a). The prognostic role of RRM2-c2orf48 was evaluated through OS estimation via Kaplan–Meier and log-rank test analyses. The median follow-up time for the entire patient set was set to 74 months. The cumulative 5-year survival rate was measured as 54.5% in the high RRM2-c2orf48 expression group, whereas it was 72.6% in the low RRM2-c2orf48 expression group (*P*<0.01, Figure 4b). Moreover, the disease-free survival rate in patients presenting high RRM2-c2orf48 protein expression was lower than the survival rate of patients presenting low RRM2-c2orf48 expression (130.3 months *versus* 141.2 months, *P*=0.022, Figure 4c). Furthermore, we subgrouped the N and M stages into two groups, and the results show that the association between high RRM2-c2orf48 expression and shorter OS is significantly stronger in patients occupying M1 stages than those occupying M0 stages (*P*<0.05, Figure 4d). However, the association between high RRM2-c2orf48 expression and shorter OS was found to be significantly stronger in patients occupying N0–1 stages than in those occupying N2–3 stages (*P*<0.05, Figure 4e).

To confirm the representativeness of the NPC cohort in this study, we tested well-established prognostic factors of patient survival. Kaplan–Meier analysis evaluated a significant impact of well- known clinicopathologic prognostic parameters, such as RRM2-c2orf48 expression (*P*=0.010), T staging (*P*=0.014), N staging (*P*=0.005), M staging (*P*<0.001) and UICC 2008 stage (*P*=0.001) on patients’ survival (Table 2). In contrast, age, gender and WHO histological classification levels had no significant impact on OS according to our univariate analysis (*P*>0.05). As a result of the multivariate analyses, M staging and clinical staging were considered to be independent and unfavorable factors (Table 2).

In conclusion, the new chimeric transcript RRM2-c2orf48 in NPC cells and tissues may be associated with tumor development.

### RRM2-c2orf48 enhances cancer cell migration and invasion

**Figure 5:**
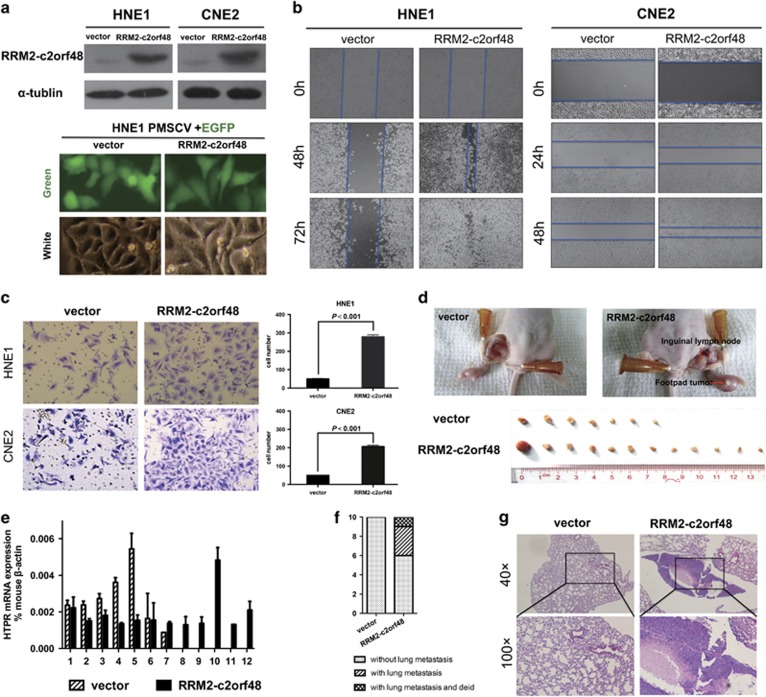
RRM2-c2orf48 enhances cancer cell migration and NPC invasion. (**a**) Establishment of stable cell lines of overexpressed RRM2-c2orf48 and confirmed by western blotting. In *vitro*, cell lines deformed to longer and thinner after overexpressed RRM2-c2orf48. (**b**) NPC cell lines of overexpressed RRM2-c2orf48 healed the wound ~72 or 48 h after serum starvation. (**c**) RRM2-c2orf48 overexpression increased the number of HNE1 cells invaded through the matrigel-covered chamber transwell. (**d**) The ratio of metastatic inguinal lymph nodes to the total number of inguinal lymph nodes dissected was markedly higher in the CNE2-RRM2-c2orf48 group (92.3%, 12/13) than in the vector-control group (53.8%, 7/13). (**e**) The ratio of HPRT in humans to *β*-actin in mice was used to confirm the existence of human cells in inguinal lymph nodes. (**f**) The ratio of lung metastases to the total number of mice was markedly higher for the CNE2-RRM2-c2orf48 group (40%, 4/10) than for the vector-control group (0%, 0/10). (**g**) H&E staining results confirm the existence of lung metastasis

There were no significant difference between vector and RRM2-c2orf48 in MTT assay and Colony formation assay (data not shown). As the acquisition of a migratory and invasive phenotype is necessary for cancer cell dissemination, we examined whether RRM2-c2orf48 regulates NPC cell migration and invasion. As shown in Figures 2a and g, NPC cell line HNE1 and CNE2 were found to include low endogenous RRM2-c2orf48 levels and were used to establish RRM2-c2orf48 stable and overexpressed cell lines, which were confirmed by western blotting at the protein levels (Figure 5a).

To investigate the functional consequences of RRM2-c2orf48 expression, wound-healing and migration assays were performed on the above cell lines. As shown in Figure 5b, RRM2-c2orf48 overexpression HNE1 and CNE2 cells healed wounds ~72 or 48 h after serum starvation, whereas the control HNE1 or CNE2 cells did not have any healing effects. RRM2-c2orf48 overexpression also increased the number of HNE1 cells invaded through the matrigel-covered chamber transwell (Figure 5c, *P*<0.05). The RRM2-c2orf48 potency of cell migration and invasion could be better than that of wild-type RRM2 and c2orf48 (Supplementary Figure 3A-B). Knockdown of RRM2-c2orf48 has been performed in C666-1 cell line. After knocking down RRM2-c2orf48, the invasive ability of C666-1 was significantly decreased (Supplementary Figure 6). These results indicate that RRM2-c2orf48 overexpression enhances NPC cell migration and invasion.

### RRM2-c2orf48 enhances resistance of chemotherapy

We have performed CHT response in RRM2-c2orf48 overexpressing cells and found RRM2-c2orf48 expressing cells developed the resistance of chemotherapy comparing to vector expressing cells (Supplementary Figure 5).

### RRM2-c2orf48 promotes NPC cell invasion and metastasis *in vivo*

The effects of RRM2-c2orf48 on cell invasion and lymph node metastasis in NPC were further investigated *in vivo* using the inguinal lymph node metastasis model. Vector-transduced or RRM2-c2orf48-overexpressing CNE2 cells were inoculated into the footpads of nude mice (*n*=13/group; Figure 5d). The resulting footpad tumors and inguinal lymph nodes were excised after 33 days and analyzed, and we found that tumors formed by RRM2-c2orf48-overexpressing CNE2 cells exhibited a more aggressive phenotype. Strikingly, the ratio of metastatic inguinal lymph nodes to the total number of inguinal lymph nodes dissected was markedly higher in the CNE2-RRM2-c2orf48 group (92.3%, 12/13) than in the vector-control group (53.8%, 7/13; Figure 5d). In addition, we found that lymph nodes in tumors formed from RRM2-c2orf48-transduced cells had larger volumes than the lymph nodes of animals injected with vector-control cells. The ratio of HPRT in humans to *β*-actin in mice was used to confirm the existence of human cells in inguinal lymph nodes (Figure 5e). As was expected, RRM2-c2orf48 overexpression resulted in a significant increase in lung metastases, and the ratio of lung metastases to the total number of mice was markedly higher in the CNE2-RRM2-c2orf48 group (40%, 4/10) than in the vector-control group (0%, 0/10; Figures 5f and g). Taken together, these results indicate that RRM2-c2orf48 promotes invasion and metastasis in NPC *in vivo*.

### RRM2-c2orf48 induces NPC cell epithelial–mesenchymal transitions and regulates signal pathways

**Figure 6:**
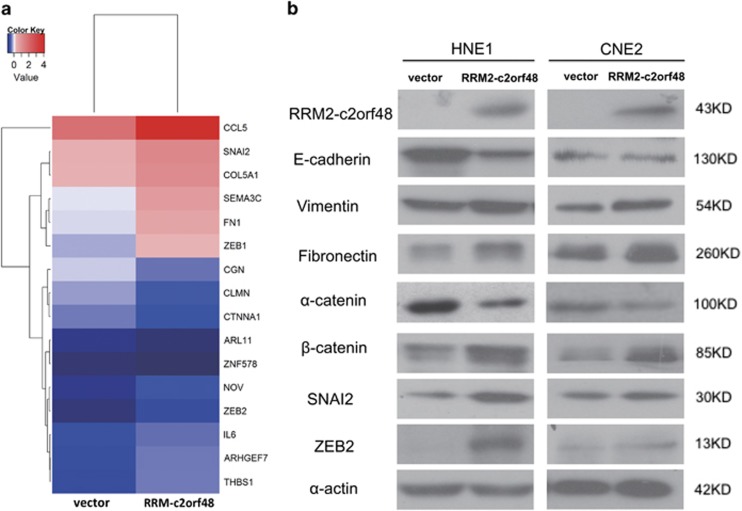
RRM2-c2orf48 induces NPC cell epithelial–mesenchymal transitioning and regulates signal pathways. (**a**) Microarray analysis of EMT-related markers. (**b**) Protein levels of EMT markers and transcript factors changed in RRM2-c2orf48 overexpressed cell lines

Recently, epithelial–mesenchymal transitioning has been found to be associated with the invasion and metastasis of malignancies. Previous study results show that chimeric protein RRM2-c2orf48 can enhance migration and invasion capacities of NPC cell lines *in vitro* and *in vivo* (Figure 5). To explore the roles of transcriptional and known markers of EMT in NPC metastasis, we generated a special gene microarray containing these elements to screen potential EMT-related genes on CNE2-PMSCV-RRM2-c2orf48 cells and paired control CNE2-PMSCV-vector cells, and the microarray data have been deposited in the Gene Expression Omnibus under accession number GSE100193. As shown in Figure 6a, 11 EMT associated genes, such as C–C motif chemokine ligand 5 (CCL5, 1.81), snail family transcriptional repressor 2 (SNAI2, 1.70), collagen type V alpha 1 chain (COL5 A1, 1.73), semaphorin 3C (SEMA3C, 1.79), fibronectin 1 (FN1, 1.67), zinc finger E-box binding homeobox 1 (ZEB1, 1.57), nephroblastoma overexpressed (NOV, 2.56), interleukin 6 (IL6, 1.65), Rho guanine nucleotide exchange factor 7 (ARHGEF7, 1.89), thrombospondin 1 (THBS1, 2.02) and zinc finger E-box binding homeobox 2 (ZEB2, 2.64), changed in CNE2-PMSCV-RRM2-c2orf48 cells relative to CNE2-PMSCV-vector cells. We then performed a western blotting test to further confirm these changed genes and EMT markers at the protein level of HNE1 and CNE2 overexpressed RRM2-c2orf48. Epithelial markers such as E-cadherin and *α*-catenin were downregulated. In contrast, mesenchymal markers such as fiberonectin and vimentin were upregulated (Figure 6b). The upregulation of SNAI2 and ZEB2 was also examined in these RRM2-c2orf48 overexpressing cells (Figure 6b). The result of GO ontology enrichments presented that most changed genes are enriched in cell differentiation, cell proliferation, biological adhesion, intracellular signal transduction and multicellular organismal development (Supplementary. Figure 7). These data suggest that RRM2-c2orf48 induces EMT and regulates related pathways in NPC.

## Discussion

NPC is an extremely narrow endemic malignant tumor form that has a high incidence in southern China. Owing to the well-developed lymphatic network in the nasopharynx, NPC has a high incidence of cervical lymph node metastasis at the point of diagnosis that is associated with a poor prognosis.^15, 16^ Therefore, there is an urgent need to identify key molecular alterations that contribute to local invasion and lymph node metastasis to provide effective targets for anti-metastatic therapy in NPC. In this study, we found that high RRM2-c2orf48 expression is significantly correlated with lymph node metastasis and poor survival rates for human NPC. Moreover, RRM2-c2orf48 causes NPC cells to invade and metastasize to lymph nodes *in vivo*. This study thus provides further insight into the mechanisms that regulate invasion and metastasis in NPC and presents RRM2-c2orf48 as a potential target of anti-metastatic therapy in treating NPC.

In this study, RRM2-c2orf48 was found to be heavily enriched in NPC. This chimera is likely generated as a result of RNA splicing rather than due to chromosomal rearrangements. The RRM2-c2orf48 transcript is described as including the former 9 exons of RRM2, 5’UTR, exon 1 and partial exon2 of c2orf48 including 5’UTR of c2orf48, thus leading to the coding of a functional chimeric RRM2-c2orf48 protein that differs from wild-type RRM2 and c2orf48. We confirm that the new chimeric transcript exists in C666-1, a NPC cell line. However, how does it expression in immortalized nasopharyngeal epithelial cell lines (NPECs) and in other NPC cells? RRM2-c2orf48 was found to be recurrent among NPC patients and enriched in tumor tissues at readily detectable levels, whereas it presents low levels of expression in noncancerous nasopharyngeal epithelial tissues. Therefore, the presence of RRM2-c2orf48 is not accidental, as it is found in C666-1 cells as well as in other NPC cells, suggesting that the new RRM2-c2orf48 chimera may be associated with the existence and progression of NPC.

Our analysis was performed on a large subset of patients and reveals the prognostic meaning of RRM2-c2orf48 expression in NPC patients. RRM2-c2orf48 expression was found to be associated with N staging and clinical staging in NPC patients. NPC cases with high RRM2-c2orf48 expression present the worst overall rates of survival and disease-free survival, suggesting the potential utility of RRM2-c2orf48 as a molecular signature for identifying NPC subgroups with metastasis.

Our previous reports have indicated that an elevated expression of parental wild-type RRM2 is associated with NPC and with other cancers.^14^ Through an *in vitro* assay, the morphology of NPC cell lines changed from a cuboidal shape to an irregular spindle shape (Figure 5a). As described in Lee MH’s study,^17^ the mechanical and adhesive properties of cancer cells change significantly during tumor progression, and individual cancer cells undergo highly transient bursts of rapid migration. Hence, it is reasonable to speculate that novel chimera RRM2-c2orf48 may be associated with cell migration and invasion potency. According to our results, RRM2-c2orf48 overexpression could enhance migration and invasion capacities of NPC cells through transwell and wound-healing assays *in vitro*. Subsequently, *in vivo* assays of RRM2-c2orf48 could induce lung and lymph node metastasis in nude mice. All of these results show that RRM2-c2orf48 could serve as an essential mediator of NPC migration and invasion.

As is well known, epithelial–mesenchymal transition (EMT) is considered to be associated with the invasion and metastasis of a malignant tumor.^18^ EMT can cause stem cells to develop NPC and immortalized breast epithelium,^19, 20, 21^ resulting in tumor metastasis and recurrence. EMT is regulated by a complex network,^22^ but the specific mechanism that controls this remains unclear. The down-expression of E-cadherin is considered as an initial EMT event. We found that RRM2-c2orf48 increases the expression of interstitial markers Vimentin and of Fibronectin but decreases epithelial markers E-cadherin and *α*-catenin, demonstrating that RRM2-c2orf48 leads to the induction of epithelial–mesenchymal transitioning in human cells. We further explored how RRM2-c2orf48 induces EMT in NPC cells. After screening potential EMTs related to microarrays and verifying them by western blotting, we found that SNAI2 and ZEB2 are upregulated in RRM2-c2orf48 overexpressing NPC cells. As has been reported in the case of NPC, Bmi-1 can increase the mobility of nasopharyngeal epithelial cells and is associated with SLUG and other transcription factors.^23^ ZEB2 is an important transcription factor among cancers,^24^ and ZEB1 promotes cancer cell dedifferentiation by repressing master regulators of epithelial polarity.^25, 26^ Therefore, increased ZEB1 and ZEB2 levels may promote EMT-dependent invasion and cancer stem cell activity, features that are essential for the metastatic spread of cancer. Herein, we showed that RRM2-c2orf48 induced EMT in NPC cells leads to the upregulation of multiple downstream genes including SNAI2, ZEB2, Vimentin, Fibronectin and *β*-catenin and to the downregulation of E-cadherin and α-catenin. Such an RRM2-c2orf48-mediated mechanism may enhance heterotypic signals induced by extrinsic stimuli to in turn induce the acquisition of mesenchymal traits by cancer cells and to promote metastasis during later stages of tumor progression. From Figure 1f, RRM2-c2orf48 does not contain 3'UTR of RRM2. So this might by-pass microRNA regulation of RRM2. According to the bioinformatics analysis from UCSC Genome Browser, there are many transcription factor (TF)-binding sites between RRM2 and c2orf48 intergenic sequence. Among these TF binding sites, some of them also exist in the promoter of RRM2, such as MYC transcription factor binding site. Therefore, the RRM2-c2orf48 chimeric transcript does not fully set c2orf48 transcription under RRM2 promoter regulation. In addition, the mRNA stabilities of RRM2 and c2orf48 were lower in CNE2-RC cell lines compared with those in CNE2-Vector cell lines. Although RRM2-c2orf48 has indeed the similar function of RRM2 or c2orf48, this novel chimeric sequence might effects the expression of RRM2 and c2orf48 to act its strong function in cell migration, invasion and EMT. However, such mechanisms require further study.

In summary, this study reveals the expression pattern of RRM2-c2orf48 in NPC and shows that high levels of RRM2-c2orf48 expression are associated with metastasis and survival in NPC patients. We also found that RRM2-c2orf48 promotes NPC cell migration and invasion. Taken together, these results suggest that RRM2-c2orf48 is a potential biomarker of metastasis and prognosis in NPC patients and may contribute to the development of NPC.

## Methods and materials

### RNA-seq experiments

Total RNA was extracted from the NPC cell line, C666-1 and NPEC2. Beads bound to oligo(dT) were used to isolate poly(A) mRNA from total RNA. cDNA libraries were prepared and sequenced on the Illumina Hi-seq2000. We used the reference sequences the genome and transcriptome sequences downloaded from the UCSC website (hg18 version). Each clean reads was aligned to the reference genome and transcriptome using SOAP2.^27^ No more than three mismatches were allowed in the alignment for each read. The fusion gene detection analyzed using the SOAPfuse software.^28^

### Tissue specimens

Sixty NPC specimens and 20 noncancerous epithelial tissues used in studies of RRM2-c2orf48 expression based on qRT-PCR were collected from the Sun Yat-sen University Cancer Center (SYSUCC) and Sun Yat-sen Memorial Hospital in Guangzhou, PR China. All biopsy specimens of qRT-PCR were immediately immersed in RNA-Later solution (AM7021, Ambion, USA) overnight at 4 °C and then preserved at −80 °C prior to RNA extraction. NPC tissue microarray analyses were performed as previously described.^29, 30^ In total, 194 cases of NPC with sufficient follow-up data were presented from the Department of Pathology, Sun Yat-sen University Cancer Center. We obtained patient consent and approval from the Sun Yat-sen University Cancer Center and Sun Yat-sen Memorial Hospital Institute Research Ethics Committee to use these clinical materials for research purposes.

### Cell lines and cell cultures

NPECs were immortalized by transfecting Bmi-1 as described previously and were cultured in keratinocyte serum-free medium (Invitrogen, Carlsbad, CA, USA)^31^ and labeled NPEC1 Bmi-1 and NPEC2 Bmi-1. NPECs N01-N09 were derived from primary cultures of fresh nasopharyngeal tissues. NPC cell lines including CNE1, CNE2, 6-10B, 5-8 F, HONE1, SUNE1 and HNE1 were obtained and cultured in RPMI-1640 medium (Gibco, USA) with 10% fetal bovine serum (Gibco) as described in our laboratory previously.^23, 31^ All of the above NPC cell lines were cultured in 37 °C with 5% CO_2_. Cisplatin (DDP) and fluorouracil (5-FU) were purchased from Sigma (Shanghai, China).

### RNA extraction and qRT-PCR analysis

Total RNA from different cell lines and human tissues was extracted using Trizol reagent (Invitrogen). RNA concentrations and quantities were determined using a NanoDrop spectrophotometer (ND-1000, Thermo Scientific, USA). After a reverse transcription of the RNA, first-strand cDNA was used as a template for detecting RRM2-c2orf48 expression. qRT-PCR and data collection were performed through a CFX96 real-time PCR detection system (Bio-Rad, CA, USA). qRT-PCR detection was normalized by an internal control, Glyceraldehyde-3-phosphate dehydrogenase (Gapdh). Primers of RRM2-c2orf48 Amplification are listed below and synthesized by Invitrogen:

RRM2-c2orf48 Sense 5′-CTTGCCTGTGAAGCTCATTGG-3′

Anti-sense 5′- CCTCACTGGCGTGCTG -3′

Gapdh Sense 5′- CTCCTCCTGTTCGACAGTCAGC-3′

Anti-sense 5′-CCCAATACGACCAAATCCGTT-3′

To ensure the reproducibility of our results, all genes were tested in triplicate. For mRNA half-life assessment in cell lines, actinomycin D (ACTD, 5 *μ*g/ml) was added after 48 h, cells were cultured and RNA was prepared at the indicated times.

### Western blotting

Cell line proteins were extracted, and a western blotting assay was performed as described above.^31^ In brief, cells were lysed in RIPA buffer containing a protease inhibitor mixture and were incubated on a rocker at 4 °C for 15 min. Lysate protein concentrations were measured using a BCA protein assay kit (Pierce) and were normalized to equal amounts of protein. The cell lysates were separated by 9% SDS-PAGE, were transferred to PVDF (Bio-Rad Laboratories) and were probed with the indicated primary antibodies. After being probed with the indicated antibodies, the blot was incubated with species-specific secondary antibodies. The same membranes were then stripped and re-probed with mouse monoclonal antibodies against *α*-tubulin or *β*-actin or*α*-tublin to ensure equal sample loading.

### Immunofluorescence assay

Cells with overexpression of RRM2-c2orf48 and vector were seeded on coverslips in 24-well plates at a density of 5 × 10^4^ cells per well for 18–24 h. They were briefly washed with PBS twice, fixed with 3% paraformaldehyde in PBS for 20 min, and then permeabilized with 0.1% Triton × 100 in PBS for 5 min. After blocking with 5% BSA in PBS, cells were stained with the indicated antibodies for 4 °C overnight and washed with phosphate-buffered saline added 1/1000 Tween-20 (PBST) three times, followed by incubation with Alexa Fluor 594-labeled secondary antibodies (1:1000 dilution). After washing with PBST three times, the cells were mounted with ProLong Gold mounting medium (Invitrogen) containing 0.2 mg/ml DAPI, which could stain nuclei. Confocal images were acquired using a Leica TCS SP5 confocal laser scanning microscope.

### Immunohistochemistry and histological evaluation

After being dewaxed in xylene and rehydrated with graded alcohol to distilled water, NPC specimen sections were immersed in 3% hydrogen peroxide for 15 min to prevent endogenous peroxidase activity at room temperature, and we then boiled the sections in antigen retrieval solution (Citrate, pH=6.0) for 4 min in a pressure cooker for antigen retrieval. After being cooled to room temperature (~26 °C for 2 h), the sections were incubated with diluted rabbit anti-RRM2-c2orf48 antibody (1:200, China Branch of ProteinTech Group, PR China) overnight at 4 °C. The next day, after being rinsed with PBST three times, the sections were incubated with the secondary antibody for 30 min at 37 °C and then rinsed with PBST three times followed by DAB (3, 3-diaminobenzidine) staining for 2 min for targeted protein identification. The sections were then counterstained with hematoxylin to stain the nucleus. After being rinsed for 2 h under flowing water and dehydrated at 37 °C, specimen sections were mounted by Neutral Balsam for preservation. For each slide, five random fields of vision were selected for scoring, and the mean score of each slide was used in our final analyses. Positive staining was assessed using a four-point scoring system: 1, negative or <25% positive tumor cells; 2, 26–50% positive cancer cells; 3, 51–75% positive cancer cells; 3, more than 76% positive cancer cells. Staining intensity levels were categorized as follows: 0, negative staining (–); 1, weak light yellow-colored staining (±); 2, (moderate yellowy brown-colored staining (+) and 3, strong brown-colored staining (++). RRM2-c2orf48 expression immunoreactivity score (IRS)=(intensity score) × (positive score), ranging from 0 to 12. All results were confirmed by at least two pathology experts through a double-blind model analysis. The final IRS (0–12) is the product of the intensity and extent scores. An optimal cutoff value for high and low expression was determined based on a log-rank test statistical analysis measurement of heterogeneity regarding the OS rate. In this RRM2-c2orf48 study, the optimal cutoff value was determined as follows: an IRS of ≤6.0 was defined as low RRM2-c2orf48 expression and an IRS of >6.0 was defined as high expression.

### Plasmids and transfection

The full-length human RRM2-c2orf48 opening reading frame (ORF) was cloned into pcDNA3.1 vector and PMSCV vector with restriction sites of *Bam*HI (or *BgI*I) and *Xho*I. Relevant primers are listed below:

Primers of full-length RRM2-c2orf48 CDS

RRM2-c2orf48 Sense (*Bam*HI) 5′-CGGGATCCATGCTCTCCCTCCGTGTC-3′

Anti-sense (*Eco*RI) 5′-CGGAATTCTTATCCCCGCACACTGGGTGG-3′

Primers of full-length RRM2-c2orf48 CDS (C-flag)

RRM2-c2orf48 Sense (*Bam*HI) 5′-CGGGATCCATGCTCTCCCTCCGTGTC-3′

Anti-sense (*Eco*RI) 5′-CGGAATTCTCCCCGCACACTGGGTGGGT-3′

The HNE1 and CNE2 NPC cell lines were infected with retroviral particles containing human chimeric gene RRM2-c2orf48 to form stable transfected cell lines. The empty vector was used as a negative control. The cells were selected and maintained in puromycin (1 *μ*g/ml).

SiRNAs targeting the mRNA of human RRM2-c2orf48 and the negtive control (Ruibo Biotechnology Company, Guangzhou, China) were transfected into NPC cells using Lipofectamine TM RNAi MAX reagent (Invitrogen) according to the manufacturer’s instructions. The siRNA sequences were as follows:

siRNA of RRM2-c2orf48 sense 5′-UGGGUUUUAGCAAGGUGCU dTdT-3′

anti-sense 5′-dTdT ACCCAAAAUCGUUCCACGA-3′

### 3-(4,5-Dimethylthiazol-2-yl)-2,5-diphenyltetrazoliumbromide (MTT) assay

To measure cell viability levels, MTT assays were conducted. Cells were seeded at 1000 cells/well in a 96-well plate in sextuplicate and allowed to attach overnight. The viability of the HNE1 or CNE2 cells was measured by incubating the cells with 0.5 mg/ml MTT every 24 h. The medium was removed, and 200 *μ*l of dimethyl sulfoxide solution was added to melt the sediment. Following vibration mixing for 10 min, the OD value was detected using a microplate reader. Three independent experiments were carried out for each assay. And the MTT assay was also used to measure the viability of the NPC cells. RRM2-c2orf48-overexpressing NPC cells were seeded onto a 96-well plate at a density of 500 cells per well. After 24 h, DDP or 5-FU at 1.5 *μ*g/ml was added into the cultures.

### Colony formation assay

Two hundred HNE1 and CNE2 cells were plated per well in triplicate in six-well plates and cultured for 10 days. After most of the colonies had expanded to >50 cells, the cells were rinsed and fixed in methanol for 15 min and dyed with crystal violet for 15 min at room temperature. Three independent experiments were carried out for each assay.

### Wound-healing assay

HNE1 or CNE2 cells were used for the wound-healing assay as described above.^23^ Three independent experiments were carried out for each assay.

### Transwell assay

HNE1 or CNE2 cells were used for the transwell assay as described above.^23^ In brief, the upper chambers with 8 mM pore polycarbonate membrane were pre-coated with Matrigel and covered with 200 *μ*l of medium without FBS, which contained 1 × 10^5^ cells. These chambers were then immersed in the lower wells, which contained 500 *μ*l of medium with 10% FBS. The cells that traversed the filters and adhered to the opposite side of the chamber membrane were photographed and counted by light microscopy after 24 h. Three independent experiments were carried out for each assay.

### Animals and Inguinal lymph node metastasis model

The inguinal lymph node metastasis model was used to investigate the role of RRM2-c2orf48 in lymph node metastasis in NPC as previously described.^32^ Five-week-old BALB/c female nude mice (18–20 g) were purchased from the Guangdong Medical Lab Animal Center Co. Ltd. and maintained in microisolator cages (Guangzhou, PR China). All experimental procedures were approved by the Institutional Animal Care and Use Committee of Sun Yat-sen University and were performed in accordance with the Declaration of Helsinki. Mice were randomly divided into two groups (*n*=13/group). CNE2-PMSCV-vector or CNE2-PMSCV-RRM2-c2orf48 cells (5 × 10^5^) were inoculated into the footpads of the mice on day 0. The mice were killed on day 33. Primary tumors and inguinal lymph nodes were excised and total RNA extracted. Hypoxanthine phosphoribosyl transferase 1 is a commonly used internal control in qRT-PCR and has been recommended as a sensitive housekeeping gene^33^ detected in the inguinal lymph nodes of mice.

### Lung metastasis model

Twenty nude mice were randomly divided into two groups (*n*=10/group), and 1 × 10^5^ CNE2-PMSCV-vector or CNE2-PMSCV-RRM2-c2orf48 stable cells were injected into their tail veins. The mice were killed at the 6th week following tumor cell inoculation. After euthanasia, the lungs of each mouse were isolated intact and fixed in 4% paraformaldehyde in PBS overnight, were embedded in paraffin, and were processed for routine histological H&E staining. H&E staining was used to distinguish cancer nests or noncancerous tissue. There were more nests in lung tissue in nude mice injected with RRM2-c2orf48 cells than in control group, in which there were no cancer nests but normal alveolar in the lung tissues.^34, 35^

### Customized gene microarray generation

A microarray analysis was performed in the vector and RRM2-c2orf48 overexpressing NPC cell samples (CNE2-PMSCV-Vector and CNE2-PMSCV-RRM2-c2orf48). Total RNA was extracted from 1 × 10^6^ cells using TRIzol reagent and was further purified using a Qiagen RNeasy Mini Kit according to the manufactures’ instructions. RNA quality was assessed by formaldehyde agarose gel electrophoresis and was quantitated spectrophotometrically. Total RNA was labeled and hybridized to the CapitalBio Corporation’s Affymetrix GeneChip Human Genome U133 Plus 2.0 Array (Affymetrix). We applied a two class unpaired method available through the Significant Analysis of Microarray software (SAM) to identify significantly differentially expressed genes between vector and overexpression samples. Genes were deemed significantly differentially expressed based on a selection threshold of a false discovery rate of FDR <5% and a fold change level of >1.5 in the SAM output results. Clustering analysis and GO term enrichment in RRM2-c2orf48 expressing cells compared with control cells were analyzed using GSEA.^36^

### Statistical analysis

The Statistical Package for Social Sciences version 16.0 (SPSS, Inc., Chicago, USA) was used to conduct the statistical analysis. The association between RRM2-c2orf48 levels and NPC patients’ clinicopathological features and correlations between detected molecular features were analyzed through a *χ*^2^- or Fischer’s exact test. Differences among variables were assessed through two-tailed Student’s *t*-tests. Survival curves were plotted through a Kaplan–Meier survival analysis and compared through log-rank testing. Univariate and multivariate regression analyses were performed using the Cox proportional hazards regression model to determine the effects of particular prognostic factors on survival. In all cases, a *P-*value of <0.05 was considered statistically significant.

## Publisher’s Note:

Springer Nature remains neutral with regard to jurisdictional claims in published maps and institutional affiliations.

## Figures and Tables

**Table 1:** Correlation between RRM2-c2orf48 expression and clinicopathologic characteristics in NPC patients

**Characteristic**	**All cases (*n*=194)**	**RRM2-c2orf48 expression (*n*=194)**		***P-*value**
		**Low (*n*=95)**	**High (*n*=99)**	
*Age (years)*				0.354
<46	80	36 (37.9%)	44 (44.4%)	
≥46	114	59 (62.1%)	55 (55.6%)	
				
*Gender*				0.887
Male	140	69 (72.6%)	71 (71.7%)	
Female	54	26 (27.4%)	28 (28.3%)	
				
*T stage*				0.001^a^
T_1–2_	88	55 (57.0%)	33 (33.3%)	
T_3–4_	106	40 (42.1%)	66 (66.7%)	
				
*N stage*				0.025^a^
N_0–1_	130	71 (74.7%)	59 (59.6%)	
N_2–3_	64	24 (25.3%)	40 (40.4%)	
				
*M stage*				0.062
M_0_	148	78 (82.1%)	70 (70.7%)	
M_1_	46	17 (17.9%)	29 (29.3%)	
				
*Clinical staging*				<0.001^a^
I–II	63	43 (45.3%)	20 (20.2%)	
III–IV	131	52 (54.7%)	79 (79.8%)	
				
*WHO type*				0.740
NKUC	145	70 (73.7%)	75 (75.8%)	
NKDC	49	25 (26.3%)	24 (24.2%)	
				
*Survival state*				0.009^a^
Survive	123	69 (72.6%)	54 (54.5%)	
Death	71	26 (27.4%)	45 (45.5%)	

Abbreviations: NKDC, non-keratinizing differentiated carcinoma; NKUC, non-kertinizing undifferentiated carcinoma.

a *P*<0.05.

**Table 2:** Univariate and multivariate Cox regression analysis of different prognostic variables in NPC patients

**Variable**	**Subset**	**Hazard ratio** **(95% CI)**	***P*-value**
*Univariate analysis (*N=*194)*			
RRM2-c2orf48 expression	High *versus* low	1.888 (1.164–3.063)	0.010^a^
Age (years)	>46 *versus* ≤46	0.767 (0.481–1.224)	0.266
Gender	Female *versus* male	1.045 (0.623–1.753)	0. 868
T staging	T1–2 *versus* T3–4	0.539 (0.330–0.882)	0.014^a^
N staging	N0–1 *versus* N2–3	0.508 (0.319–0.811)	0.005^a^
Metastasis	Yes *versus* No	2.711 (1.677–4.381)	<0.001^a^
UICC 2008 stage	I–II *versus* III–IV	0.350 (0.188–0.651)	0.001^a^
WHO histological classification	NKDC *versus* NKUC	0.563 (0.303–1.048)	0.070
			
*Multivariate analysis (*N=*56)*			
Metastasis	Yes *versus* no	2.043 (1.221-3.418)	0.007^a^
UICC 2008 stage	I–II *versus* III–IV	0.460 (0.236-0.896)	0.022^a^

a *P*<0.05.
