# Supplementary figures and images for: Novel chimeric transcript RRM2-c2orf48 promotes metastasis in nasopharyngeal carcinoma
Source: Cell Death Dis. 2017 Sep 14;8(9):e3047–. doi: 10.1038/cddis.2017.402 (PMC5636969; doi:10.1038/cddis.2017.402)

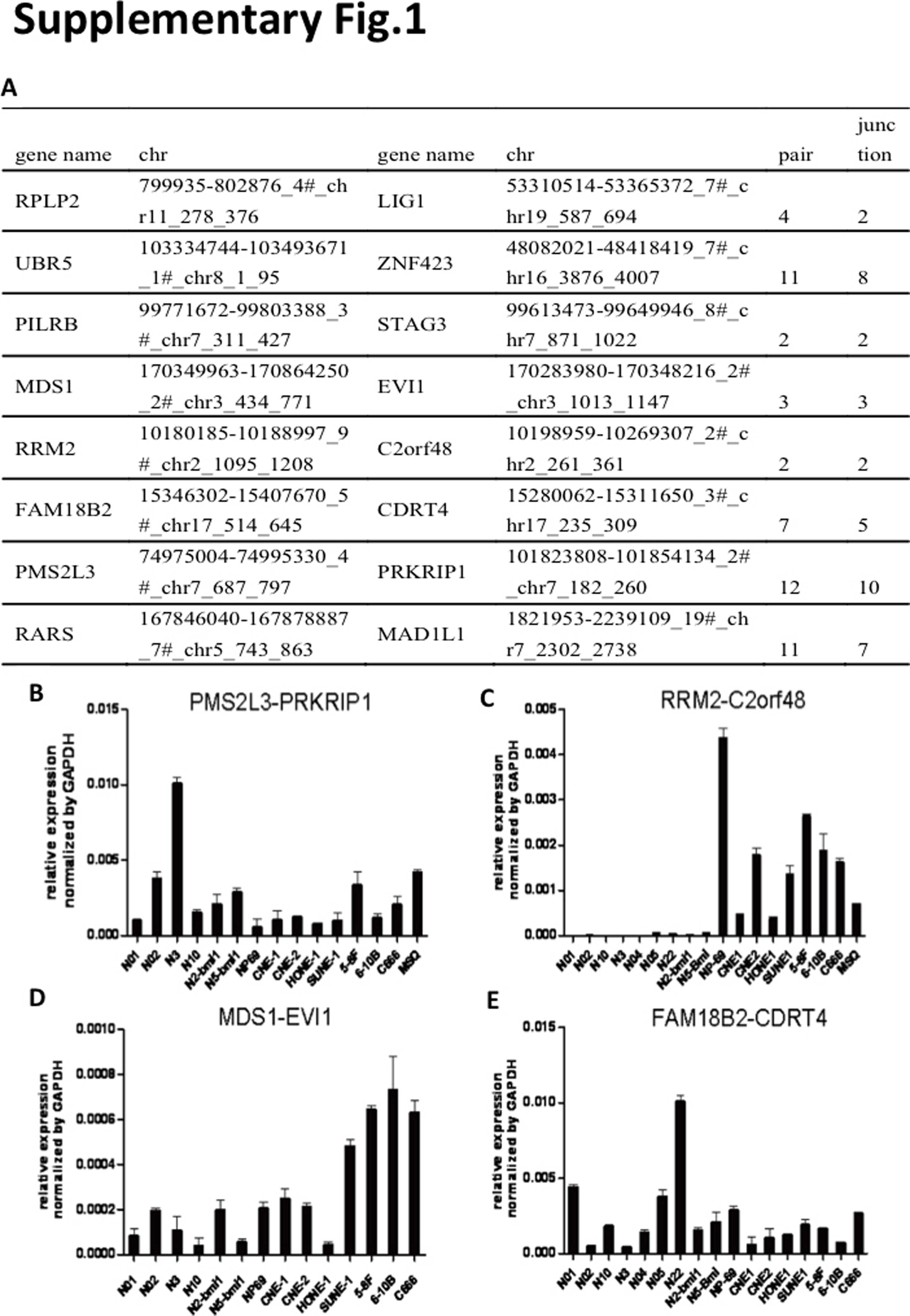

Supplement: Supplementary Figure 1 [file cddis2017402x1.tif]

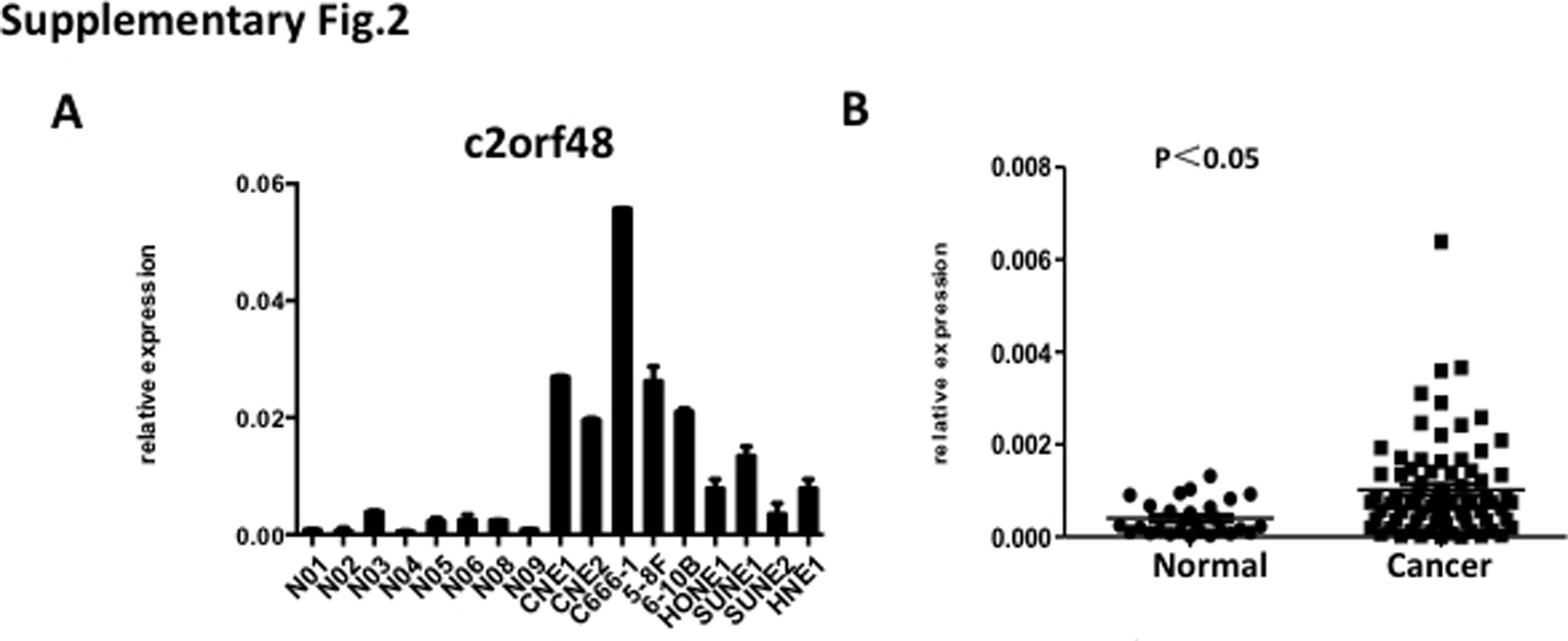

Supplement: Supplementary Figure 2 [file cddis2017402x2.tif]

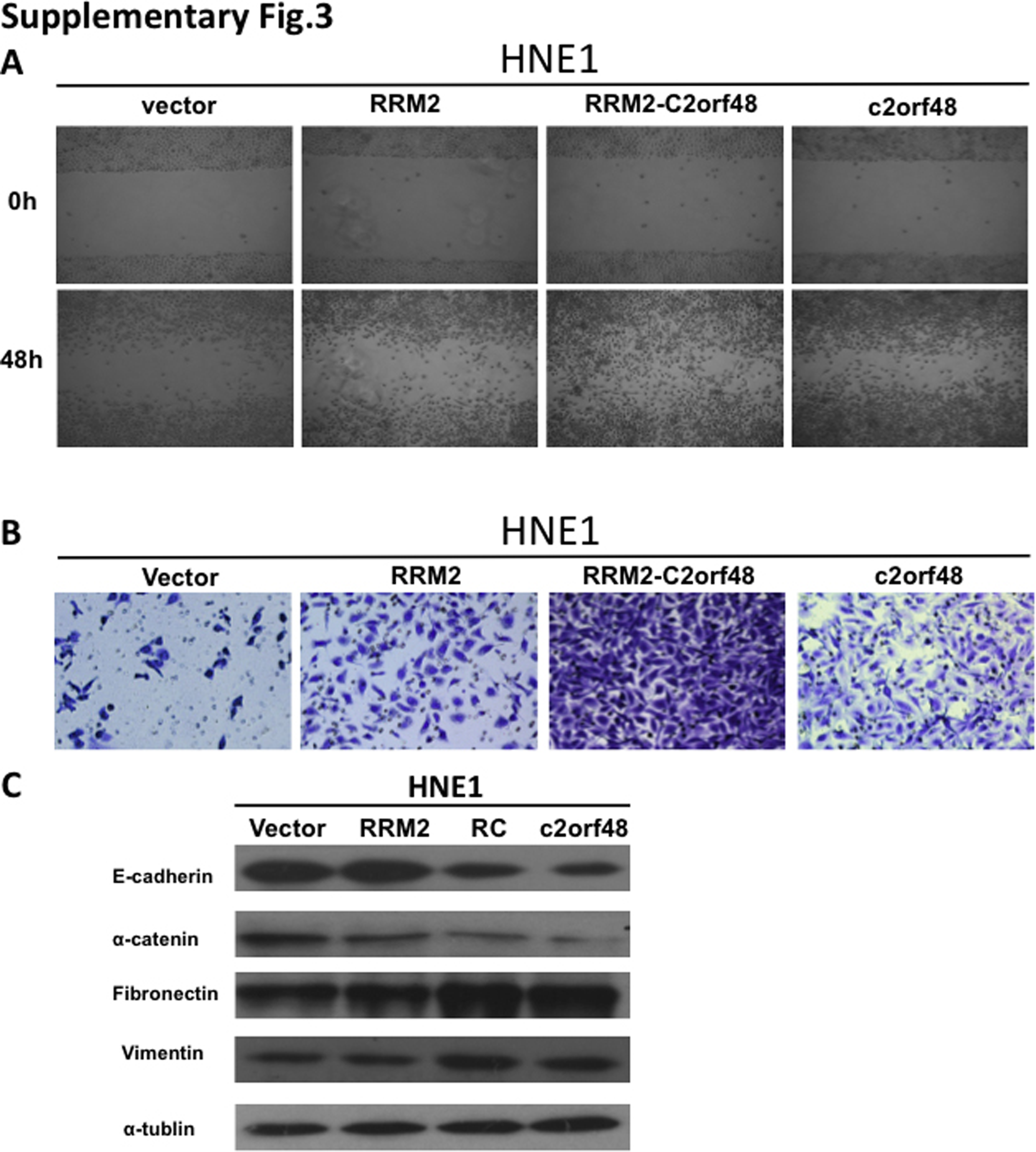

Supplement: Supplementary Figure 3 [file cddis2017402x3.tif]

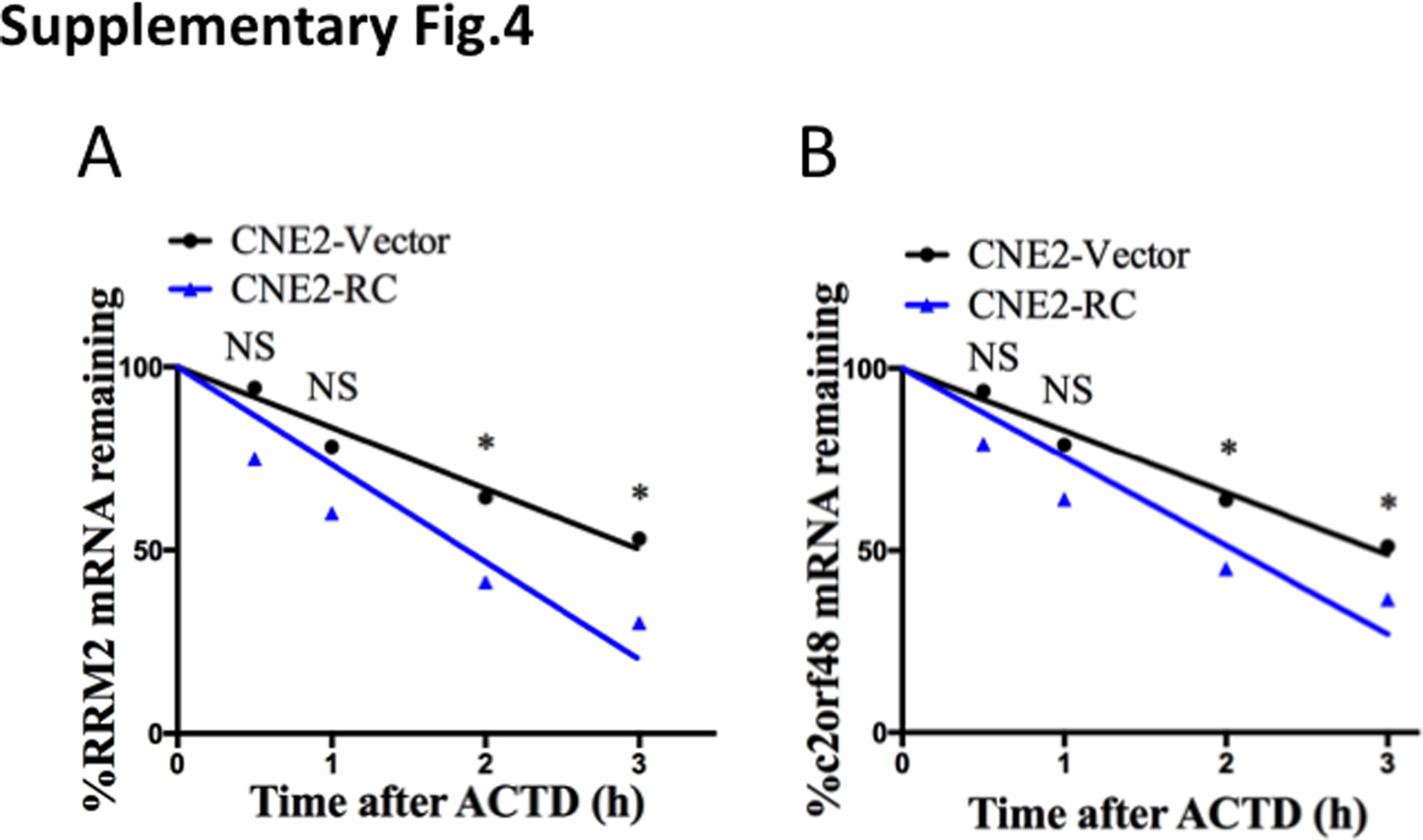

Supplement: Supplementary Figure 4 [file cddis2017402x4.tif]

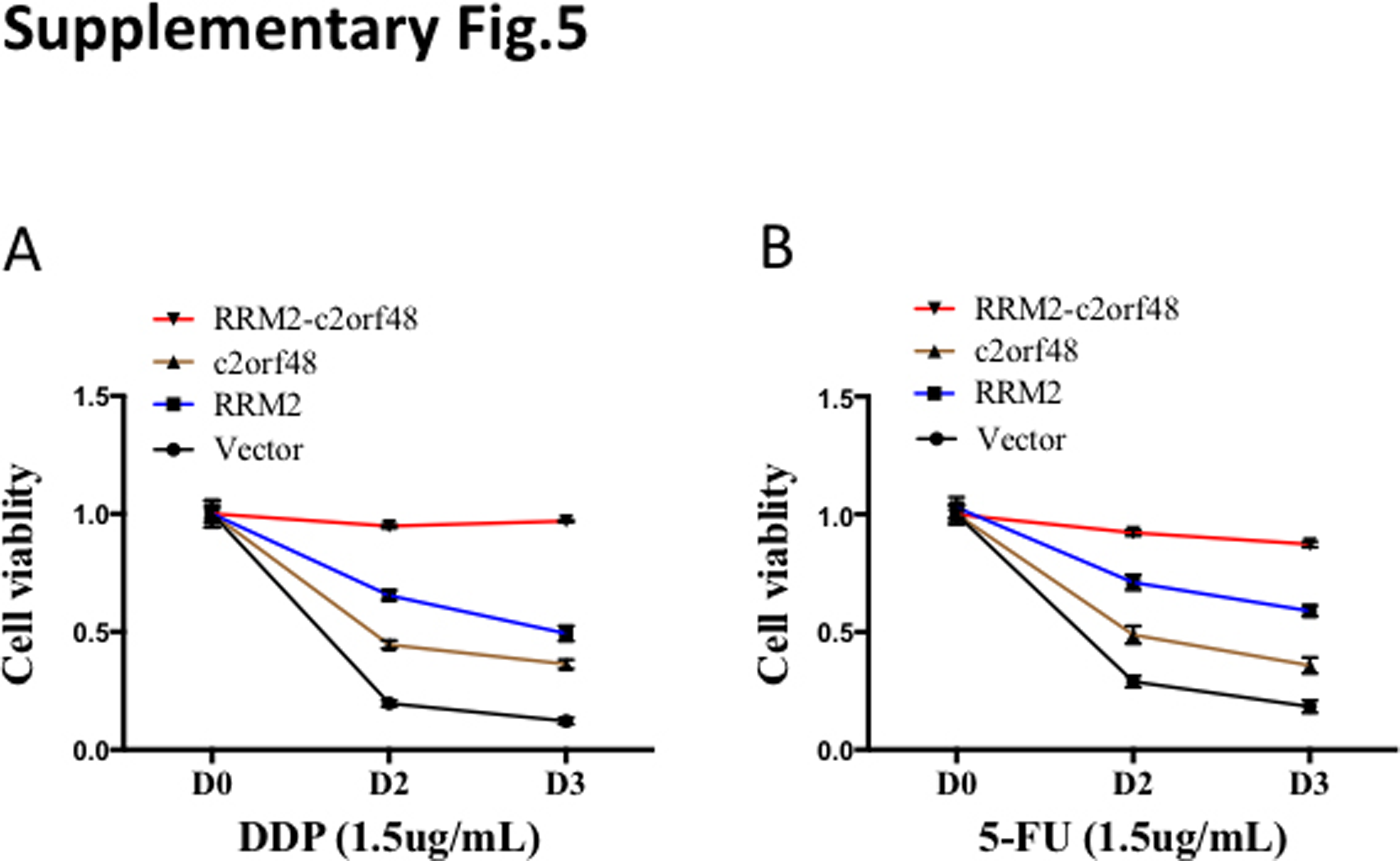

Supplement: Supplementary Figure 5 [file cddis2017402x5.tif]

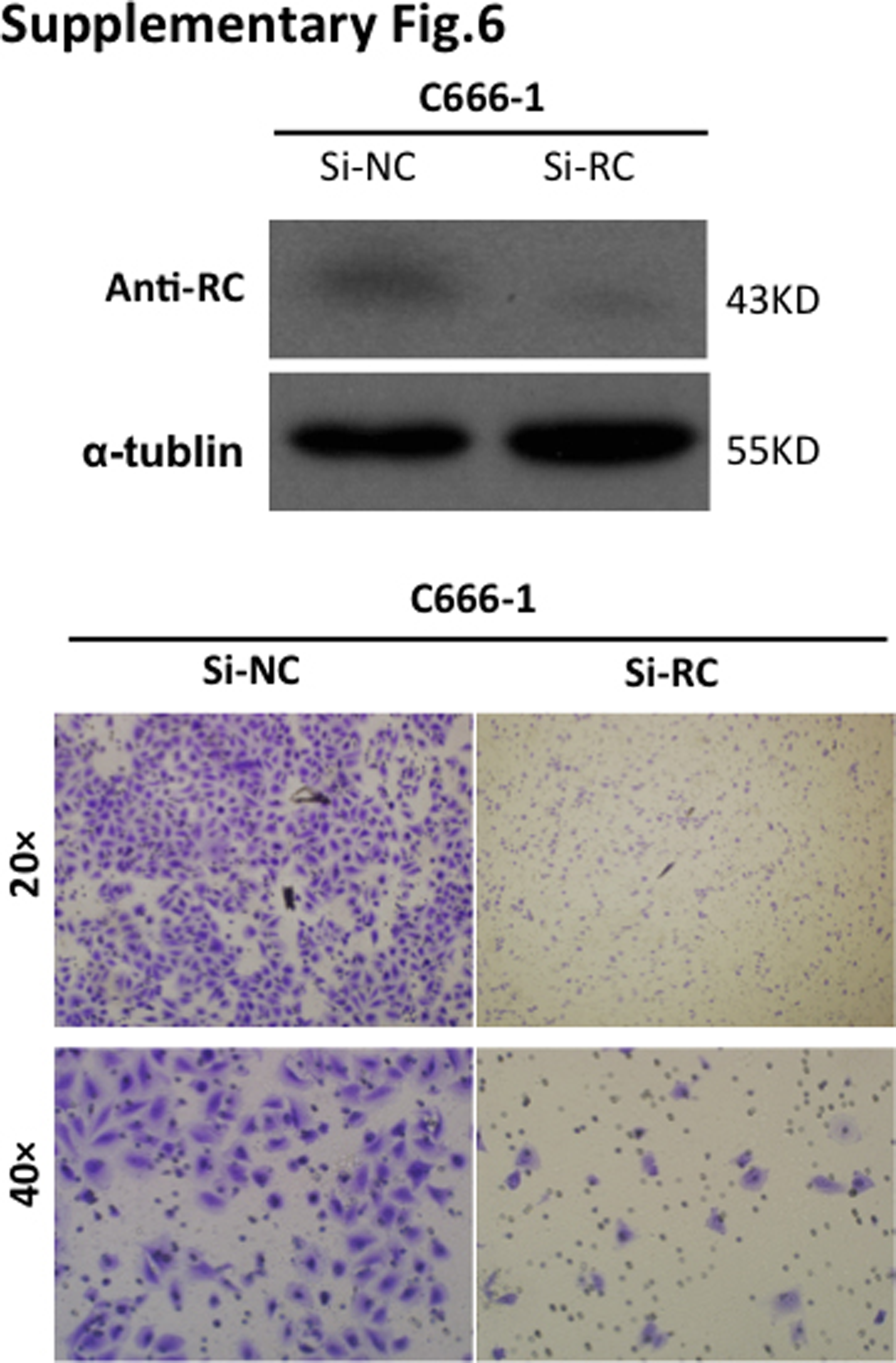

Supplement: Supplementary Figure 6 [file cddis2017402x6.tif]

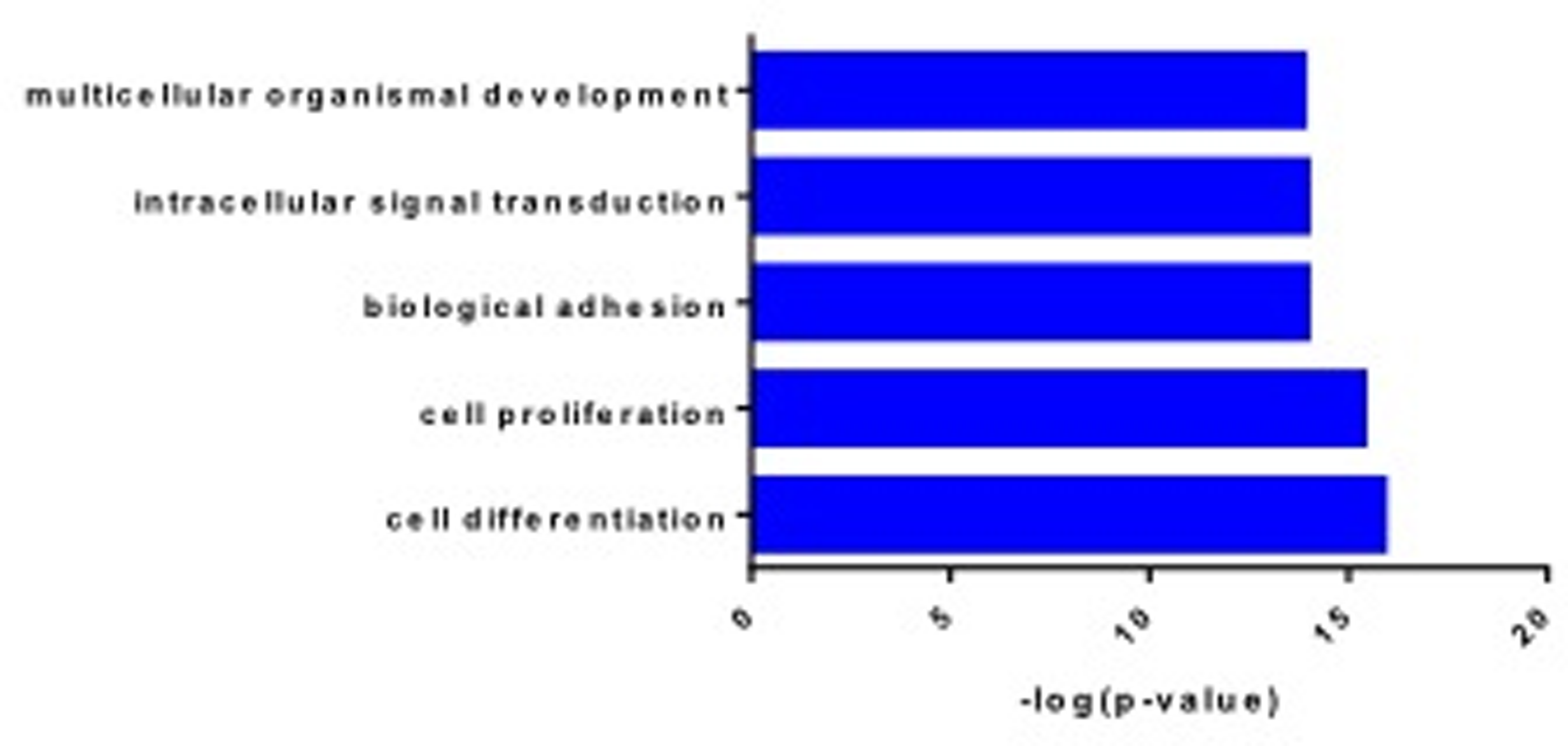

Supplement: Supplementary Figure 7 [file cddis2017402x7.tif]
