# Supplementary material for: Novel chimeric transcript RRM2-c2orf48 promotes metastasis in nasopharyngeal carcinoma
Source: Cell Death Dis. 2017 Sep 14;8(9):e3047–. doi: 10.1038/cddis.2017.402 (PMC5636969; doi:10.1038/cddis.2017.402)
Supplement: Supplementary Figure Legends [file cddis2017402x8.docx]

**Supplementary Figure Legends**

**Supplementary Fig.1 8 Different chimera candidates were found in C666-1**

**(A)** Information of 8 chimera candidates was shown, including 4 interchromosome chimeric transcripts, which was detected by qPCR:

**(B)** PMS2L3-PRKRIP1

**(C)** RRM2-c2orf48

**(D)** MDS1-EVI1

**(E)** FAM18B2-CDRT4

**Supplementary Fig.2 Expression of wild type c2orf48 in NPC**

**(A)** The expression level of wild type c2orf48 mRNA in nasopharyngeal carcinoma cell lines (CNE1 to HNE1) was higher than that in normal nasopharyngeal epithelium cells (N01-09) and in immortalized nasopharyngeal cell lines (NPEC1-2 Bmi-1) by real‑time PCR.

**(B)** The expression level of wild type c2orf48 mRNA in nasopharyngeal carcinoma specimens was higher than that in the control specimens by real‑time PCR (P＜0.03).

**Supplementary Fig.3 Function of wild type RRM2, c2orf48 and RRM2-c2orf48 in NPC**

**(A)** NPC cell lines of overexpressed RRM2-c2orf48 healed the wound approximately 48 hours after serum starvation; however, cell lines of overexpressed wild type RRM2 and c2orf48 could not healed so far.

**(B)** RRM2-c2orf48 overexpression increased more cells invaded through the matrigel-covered chamber transwell than wild type RRM2 and c2orf48.

**(C)** HNE1 overexpressed RRM2-c2orf48 could induce NPC cell EMT and the protency was stronger than that of wild type RRM2 and c2orf48.

**Supplementary Fig.4 RRM2 and c2orf48 mRNA stability is enhanced by RRM2-c2orf48 overexpression**

(A) Increase of RRM2 mRNA half-life by RRM2-c2orf48 overexpression (P=0.003). The levels of RRM2 mRNA in cells of CNE2 Vector and RRM2-c2orf48 were measured following treatment with actinomycin D for 0.5, 1, 2 and 4 h.

(B) Increase of c2orf48 mRNA half-life by RRM2-c2orf48 overexpression (P=0.006). The levels of c2orf48 mRNA in cells of CNE2 Vector and RRM2-c2orf48 were measured following treatment with actinomycin D for 0.5, 1, 2 and 4 h.

**Supplementary Fig.5 RRM2-c2orf48 could enhance resistance of chemotherapy in NPC cells**

(A) RRM2-c2orf48 could enhance resistance to DDP 1.5ug/ml in CNE1 cells.

(B) RRM2-c2orf48 could enhance resistance to 5-FU 1.5ug/ml in CNE1 cells.

**Supplementary Fig.6 Si RRM2-c2orf48 coud decrease the invasive ability of C666-1**

**(A)** RRM2-c2orf48 was knockdown in C666-1 by si RNA.

**(B)** Si RRM2-c2orf48 decreased cells invaded through the matrigel-covered chamber transwell**.**

**Supplementary Fig.7 The result of GO ontology enrichments of microarray in CNE2**

The GO ontology enrichments of the genes showing altered expression (fold change >1.5) in RRM2-c2orf48 overexpressed cell line compared with control cell line.
